# Improving the validity of breast density assessment

**DOI:** 10.1186/bcr2976

**Published:** 2011-11-04

**Authors:** A Eadie, P Whelehan, L Baker, J Berg, A Evans

**Affiliations:** 1 University of Dundee, UK

## Introduction

As the importance of mammographic density in risk stratification and breast cancer research continues to grow, density assessment must be rigorous. Area-based human visual density assessment will continue until software is fully validated. Locally, having reviewed the literature, we chose two tools and sought to establish that these would give meaningful results in our hands for comparison with other data.

## Methods

In 50 mammograms, four observers each estimated the percentage dense area, recorded on a 100 mm visual analogue scale (VAS). On a separate occasion, each observer also assigned BIRADS density scores. The process was repeated with a minimum interval of 1 week. Observers were blinded to each others' scores and their own previous scores. Viewing parameters were standardised.

## Results

### BIRADS

Intra-rater agreement: Observers 1, 2 and 4, intraclass correlation coefficient (ICC) and lower 95% confidence bound all above 0.8 (excellent agreement); below 0.8 for Observer 3. Inter-rater: Observers 2 and 4, ICC = 0.97, 95% CI = 0.95 to 0.98; Observers 1 and 3 with any other, ICC below 0.8.

### Percentage

Intra-rater agreement, ICC for all observers >0.9 with lower confidence bound >0.8. Inter-rater: Observers 2 and 4 and 2 and 3, ICC and lower confidence bound >0.8; all other pairs, <0.8.

## Conclusion

Intra-observer and inter-observer agreement in mammo-graphic density assessment varies. Percentage dense area estimation using a VAS appears more reproducible than the BIRADS classification. This simple study enabled selection of the most reliable observers and we recommend that other centres undertaking scientific studies where mammographic density is a relevant variable perform similar audits to maximise outcome measure validity.

